# Recent Advances in Pulsed Laser Deposition of REBa_2_Cu_3_O_7−δ_ High-Temperature Superconducting Coated Conductors and Artificial Flux Pinning

**DOI:** 10.3390/ma18214988

**Published:** 2025-10-31

**Authors:** Ziheng Guo, Liangkang Chen, Yuxiang Li, Xinyue Xia, Guangyao Lin, Penghong Hu, Dongliang Gong, Dongliang Wang, Yanwei Ma

**Affiliations:** 1 Key Laboratory of Applied Superconductivity, Institute of Electrical Engineering, Chinese Academy of Sciences, Beijing 100190, China; 2 School of Electronic, Electrical and Communication Engineering, University of Chinese Academy of Sciences, Beijing 100049, China; 3 Institute of Electrical Engineering and Advanced Electromagnetic Drive Technology, Qilu Zhongke, Jinan 250013, China

**Keywords:** REBCO coated conductors, high-temperature superconductors, pulsed laser deposition, artificial pinning centers, critical current density (*J*_c_)

## Abstract

Rare-earth barium copper oxide (REBCO) high-temperature superconductors, owing to their ability to maintain high critical current density (*J*_c_) under liquid-nitrogen-temperature and high-magnetic-field conditions, are widely regarded as one of the most promising material systems among all superconductors. This review systematically summarizes fabrication strategies for REBCO coated conductors, with a focus on pulsed laser deposition (PLD) for achieving high-quality epitaxial growth with precise composition control. To enhance in-field performance, strategies for introducing artificial pinning centers (APCs) are examined, including rare-earth element doping, substrate surface decoration, and nanoscale secondary phase incorporation. The mechanisms of vortex pinning from different dimensional defects and their synergistic effects are compared. Finally, we suggest potential future directions aimed at further enhancing the superconducting properties.

## 1. Introduction

**Figure 1:**
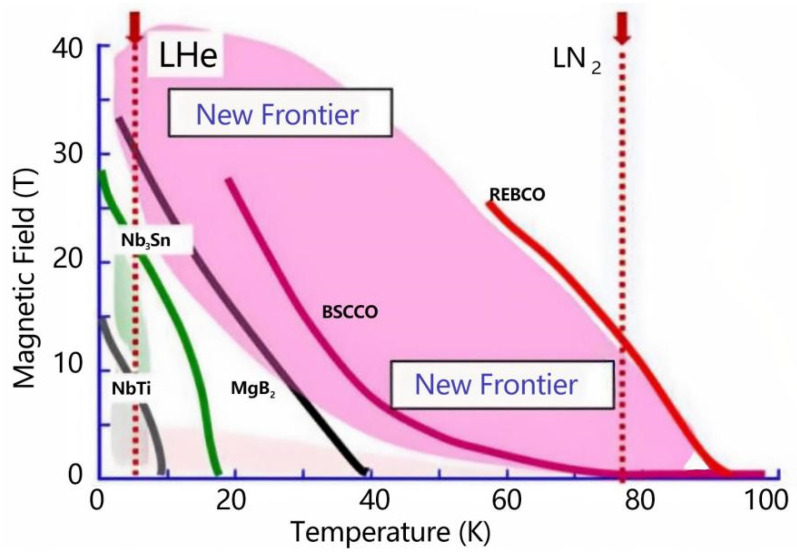
Variation of irreversibility field with temperature for different superconductors [10].

High-temperature superconductors (HTS) show tremendous potential for high-field magnet applications and efficient power transmission systems [1], and developing high-performance HTS materials has been a long-standing goal in the scientific community. HTS tapes have broad application prospects in power transmission, magnetic resonance imaging (MRI), particle accelerators, and maglev trains, and are expected to become a key component of large-scale superconducting applications in the future [2,3,4,5,6,7,8]. Compared to Bi-based HTS materials, rare-earth barium copper oxide (REBCO, where RE denotes rare-earth elements such as Y, Sm, Gd, Eu, etc.) is currently the only high-temperature superconductor capable of operating in high-field environments at liquid-nitrogen temperatures (Figure 1) [9,10,11], offering significant advantages for efficient power delivery and high-field magnet technologies [12].

Achieving the superior current-carrying capability of REBCO requires precise control of grain orientation via biaxially textured substrates combined with epitaxial thin-film growth techniques. This approach effectively suppresses weak-link grain boundaries and optimizes anisotropy [13]. The fabrication process of REBCO thin films directly determines their microstructural quality and superconducting performance. The primary fabrication methods include metal organic deposition (MOD) [14], pulsed laser deposition (PLD) [15], reactive co-evaporation (RCE) [16], and metal organic chemical vapor deposition (MOCVD) [17]. Among these, PLD is a leading-edge technology in materials science, demonstrating outstanding performance in HTS research. Through precise ablation of solid targets using high-energy laser pulses, PLD enables accurate control over film composition and thickness, and has been widely adopted for the scalable production of high-quality REBCO thin films [18]. Further enhancement of the in-field performance of REBCO in high magnetic fields can be achieved by introducing artificial pinning centers (APCs) to strengthen vortex pinning, thereby increasing the critical current density and reducing its anisotropy—an active research focus in recent years [19,20,21].

This review provides a comprehensive overview of performance optimization strategies for REBCO coated conductors, with particular emphasis on the principles, advantages, and developments of PLD. Additionally, it discusses various APC design and implementation methods, including rare-earth element doping, substrate surface decoration, and nanoscale secondary phase incorporation, aiming to offer insights for the engineering of REBCO tapes in high-field applications.

## 2. Fabrication Technologies of REBCO High-Temperature Superconducting Tapes

Due to the REBCO material is highly anisotropic and extremely sensitive to grain misorientation [1]. Randomly oriented grains lead to very weak or no intergranular current and thus cannot be fabricated into tapes using traditional Powder-in-Tube (PIT) processes like Bi-based materials [22]. Therefore, the biaxially textured epitaxial growth techniques were developed for REBCO superconductors, which are classified as second-generation (2G) high-temperature superconducting tapes, also known as coated conductors [23]. REBCO coated conductors typically employ a multilayer structure design consisting of (from bottom to top): metal substrate, buffer layer, REBCO superconducting layer and protective layer [24,25]. The metal substrate, usually made of Hastelloy or stainless steel, provides mechanical strength and flexibility as the structural support of the entire conductor; the buffer layer (such as MgO, CeO_2_, ABAD-Y_2_O_3_-Stabilized ZrO_2_ (YSZ), etc.) achieves biaxial texture through physical or chemical methods, serving the functions of lattice matching, suppressing elemental diffusion and promoting epitaxial growth of the superconducting layer; the REBCO superconducting layer is the core functional layer with high critical current density and excellent high-field performance that determines the superconducting properties of the conductor; the top protective layer (such as silver or copper cladding) protects the superconducting layer from oxidation while providing electrical stability and connection functions in applications [26,27]. The overall manufacturing system of superconducting tapes largely depends on the synergistic optimization of two key technologies: one is the texture control technology that constructs ideal crystal orientation through buffer layers, and the other is the growth process that achieves high-quality superconducting film deposition, both of which jointly determine the final tape performance and feasible fabrication routes [28].

The main techniques for preparing biaxial texture include: rolling-assisted biaxially textured substrates (RABiTS), ion beam-assisted deposition (IBAD), and inclined substrate deposition (ISD) [29]. The RABiTS technique induces highly textured recrystallized grains on metal tapes by performing multiple rolling and heat treatment processes on flexible metals (such as Ni alloys), thereby providing an orientation template for subsequent epitaxial growth [30,31]. The IBAD technique forms a biaxially oriented buffer layer by ion beam bombardment of deposited materials (such as MgO) on non-textured metal substrates, offering advantages of high process stability and strong adaptability [32]. The ISD technique controls the angle between the inclined substrate and the evaporation source to give deposited particles directionality, thereby inducing a certain degree of textured growth [33,34]. All three techniques can provide the foundation for epitaxial growth of REBCO superconducting layers, but each has distinct characteristics in terms of texture quality, production efficiency, and cost. Currently, IBAD technology represents the main direction for future development [35].

The preparation of superconducting layers in REBCO coated conductors is a critical factor affecting their performance, with fabrication methods including physical vapor deposition (PLD, sputtering, PED, etc.), chemical vapor deposition (CVD, MOCVD), chemical solution methods (e.g., metallorganic deposition/MOD, spray-pyrolysis, etc.), reactive co-evaporation (RCE), and liquid phase epitaxy (LPE) [14,15,16,17]. Among these, LPE and spray-pyrolysis have proven difficult to apply [36], while PLD, MOCVD, RCE, and MOD have developed into highly effective and commonly used methods. These four techniques each have distinct advantages and disadvantages. PLD technology is widely employed in laboratories and small-scale production due to its precise control over film composition and thickness, as well as its suitability for high-quality deposition of multicomponent oxides, but suffers from low deposition rates and high equipment costs [13]. The MOD method offers advantages of simple equipment and low cost, making it suitable for large-area preparation, but produces films with inferior crystalline quality and requires stringent control over deposition atmosphere and temperature, which can easily affect film uniformity [37]. The MOCVD method provides high deposition rates and enables uniform film growth, particularly suitable for large-scale production of REBCO films, yet it involves complex equipment and high costs and requires high-purity metalorganic precursors [38]. Additionally, the RCE method is well-suited for large-area film growth with good material quality and high growth rates but demands high temperatures and precise reaction control during operation while being limited by substrate selection [39]. Overall, PLD and MOCVD demonstrate superior performance in film quality and composition control, whereas MOD and RCE offer greater advantages in terms of cost and production efficiency, though they are slightly inferior in film quality and control precision.

Currently, various research institutions worldwide have achieved kilometer-scale superconducting tapes through different technological approaches and realized commercial sales, with the critical current per unit width *I*_c_ (77 K, self-field) generally exceeding 380 A/cm-w. Meanwhile, manufacturers are actively striving to further enhance the performance of REBCO tapes under high-magnetic-field conditions [1]. Table 1 [19,40,41,42,43,44,45] summarizes the technological approaches, compositional structures, and superconducting current-carrying capabilities adopted by major industrial enterprises worldwide.

## 3. PLD Method: Key Principles, Advantages, and Development

Pulsed laser deposition (PLD) is a thin film fabrication technique that combines high-energy-density lasers with thin film growth processes, occupying a central position in high-temperature superconducting material research and applications due to its outstanding composition transfer capability, process controllability, and unique advantages for complex oxide systems [13]. Particularly in the preparation of REBCO (rare-earth barium copper oxide) superconducting tapes, PLD has become one of the most mature and widely used methods because it enables high-quality epitaxial growth and precise control of film structure and stoichiometry [46].

**Figure 2:**
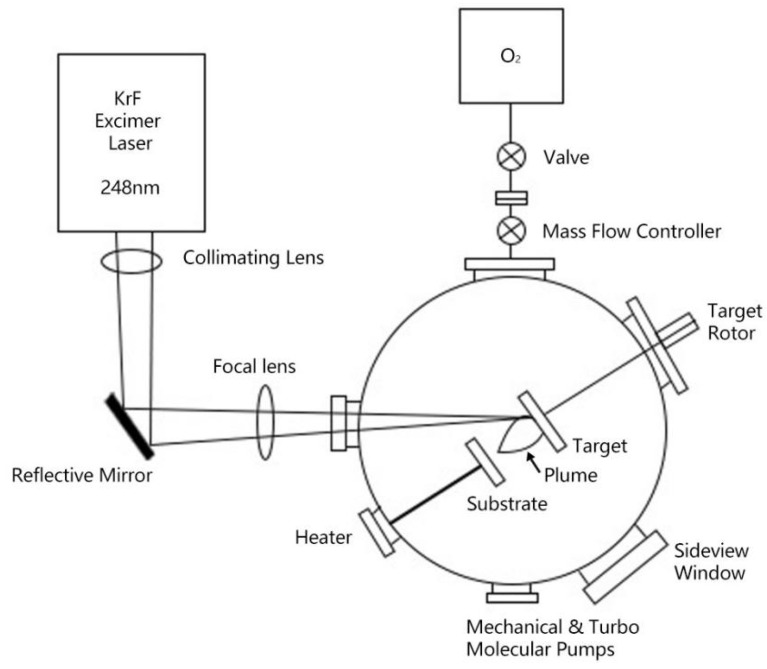
Schematic diagram of PLD system [48].

The fundamental principle of PLD relies on high-energy laser pulses to ablate a target material, generating a plasma plume within an extremely short time period and efficiently transferring the elements from the target to a heated substrate surface under controlled ambient gas and temperature conditions, thereby achieving high-quality thin film deposition [46], as illustrated in Figure 2 [47]. Compared to other deposition methods, PLD possesses several outstanding advantages: first, its concentrated energy enables efficient ablation of multi-element targets, and with proper parameter control, it can achieve nearly ideal stoichiometric transfer; second, the deposition process can be highly instantaneously controlled, facilitating thickness adjustment and fine structural regulation; third, it can operate in various reactive atmospheres such as oxygen and nitrogen, accommodating the growth environment of complex oxides like REBCO [47,48,49]. These characteristics make PLD an ideal platform for exploring and preparing high-performance REBCO superconducting films.

In the preparation of REBCO tapes, the parameters of the PLD process are crucial in determining the final film performance [13]. Firstly, the laser energy density (fluence) directly affects the target ablation efficiency and the characteristics of the plasma plume, thereby influencing the film formation rate and uniformity—insufficient energy density may lead to component deficiency, while excessive energy can cause particle splashing and compositional segregation [46]. Secondly, the ambient gas pressure (particularly oxygen partial pressure) controls the plasma transport behavior and the oxidation degree of the film, serving as a key factor determining the superconductivity and structural integrity of REBCO [22]. Additionally, the substrate temperature significantly impacts the crystalline quality, orientation, and interfacial stress of REBCO films, typically maintained between 750 °C and 900 °C, as temperatures too low will hinder grain growth while excessive temperatures may cause element loss or structural damage [50]. Therefore, precise coordination of these parameters forms the foundation for achieving high-performance REBCO film preparation.

**Figure 3:**
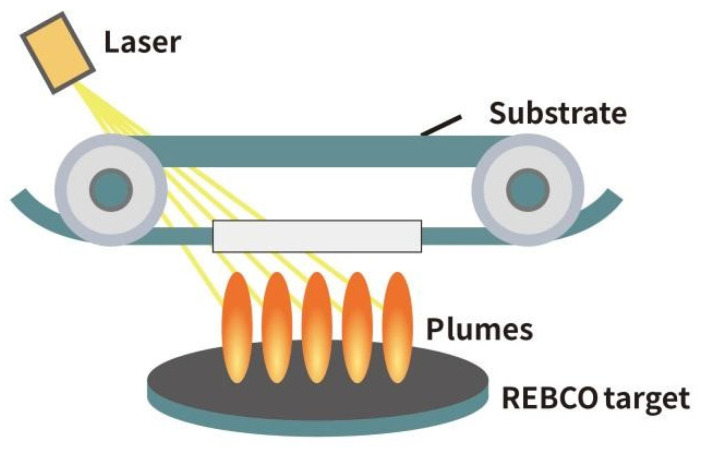
Schematic diagram of multi-plume and multi-turn PLD system.

With the widespread application of PLD technology in REBCO superconducting film preparation, its equipment systems have gradually evolved from laboratory-scale setups to industrial platforms [51]. Early PLD systems were primarily designed for basic scientific research, limited to small-area single-crystal film preparation [52,53]. In recent years, driven by the application demands of REBCO in energy, transportation, and magnet technology, PLD systems have undergone significant upgrades in structure and functionality [54,55]. The introduction of multi-target rotation systems and multiple plumes has significantly improved film thickness uniformity and deposition area [56]; roll-to-roll PLD (Figure 3) has enabled the tape to travel along its length at a controlled speed, facilitating dynamic deposition of the REBCO film and significantly increasing the deposition rate [57]. The roll-to-plume PLD system is now widely adopted for the industrial-scale, kilometer-length production of REBCO tapes [58].

It is worth noting that there are considerable differences between laboratory-scale preparation and industrial-scale production. Laboratory PLD systems typically operate at deposition rates below 1 nm·s^−1^ and mostly under static conditions, where the plume dynamics and substrate temperature are relatively ideal. These conditions enable precise control over film stoichiometry, interfacial strain, and the morphology of artificial pinning centers [59]—a topic that will be discussed in detail in the following section. In contrast, industrial production prioritizes higher throughput and productivity, typically employing high-power excimer lasers and faster tape translation speeds to achieve ultrafast growth of REBCO films, with deposition rates generally ranging from 50 to 100 nm·s^−1^. Notably, Shanghai Superconductor Co. (Shanghai, China). has reported peak deposition rates exceeding 100 nm·s^−1^, with tape speeds greater than 100 m/h [60], enabling daily outputs of more than 800 m of REBCO tapes [61] to meet large-scale production demands. Under such dynamic conditions, the behavior of the plasma plume, the mobility of adsorbed atoms, and the local crystallization kinetics are significantly altered, leading to variations in the density and orientation of artificial pinning centers (APCs), which in turn directly affect the performance of the REBCO tapes [62,63]. Moreover, maintaining stable performance over kilometer-scale lengths, ensuring tape uniformity, and minimizing batch-to-batch variability are critical issues that must be addressed in industrial-scale production. Therefore, processing strategies that are effective under conventional laboratory conditions often require further optimization before being implemented for long-length tape fabrication [63].

Although PLD-based REBCO fabrication has successfully achieved industrialization owing to its reliability and reproducibility, the high cost of PLD equipment and the limitations in production scale result in a manufacturing cost that remains considerably higher than that of low-temperature superconductors and conventional copper wires (≥100 $/km) [1,63]. To achieve large-scale commercialization, the production cost must be reduced to approximately 25–50 $/km, which would enable REBCO conductors to gain broader market acceptance in power, transportation, and high-field applications while meeting economic feasibility requirements [64]. Therefore, it is essential not only to further reduce the cost of PLD equipment and optimize production lines but also to enhance the superconducting performance of REBCO tapes to improve their overall cost-effectiveness.

## 4. Flux Pinning and Natural Pinning Centers in REBCO

In Type-II superconductors such as REBCO coated conductors, magnetic flux penetrates the material in the form of quantized vortices, each carrying a single flux quantum $\Phi_{0}=h/2e$ [65,66]. When a transport current flows through the superconductor, these vortices experience a Lorentz force density:

*F*_L_ = *J* × *B*, (1)

which drives them perpendicular to both the current density *J* and the magnetic induction *B*. The resulting vortex motion produces energy dissipation and resistivity [67].

**Figure 4:**
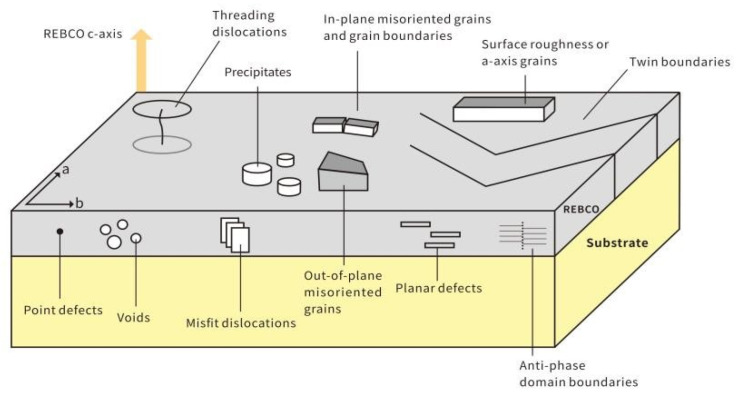
Thin-film defects have been proposed as flux pinning sites in REBCO.

To sustain dissipation-free current flow, vortex motion must be suppressed by structural inhomogeneities such as dislocations, nanoparticles, and strain fields—collectively known as pinning centers [68]. These defects exert a counteracting pinning force *F*_p_, representing the restoring force density that immobilizes vortices. The macroscopic pinning force is quantitatively defined as:

*F*_p_ = *J*_c_ × *B*, (2)

where *J*_c_ is the critical current density and *B* is the magnetic flux density. The critical current density corresponds to the threshold where the Lorentz force exceeds the maximum pinning force that can be sustained by the defect landscape. Figure 4 demonstrates how defects such as dislocations and crystallographic defects serve as pinning centers, providing localized barriers to vortex motion. The pinning force is a function of the type, distribution, and density of these defects [69].

**Figure 5:**
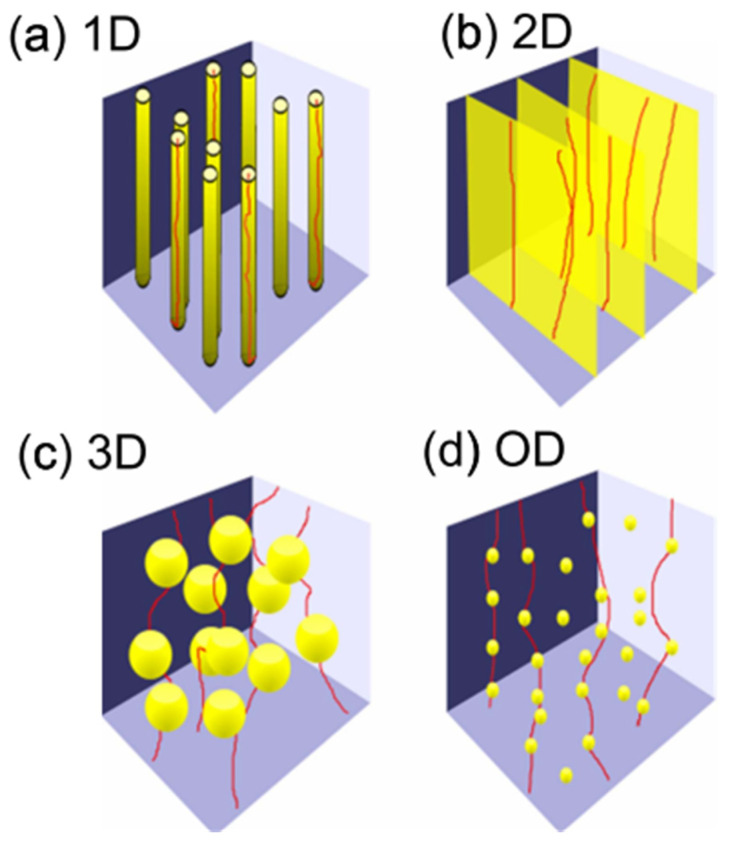
Schematic illustration of flux lines (red) interacting with pinning defects of different dimensionalities (yellow): (**a**) 1D linear defects, (**b**) 2D planar defects, (**c**) 3D large-scale random defects, (**d**) 0D point-like defects [70].

Based on the size and spatial characteristics of defects, these pinning centers can generally be classified into one-dimensional (1D), two-dimensional (2D), three-dimensional (3D) defects, and zero-dimensional (0D) defects, as shown in Figure 5 [70]. Each type of defect plays distinct roles in flux pinning mechanisms and application scenarios. One-dimensional defects exhibit pronounced directional characteristics, commonly including threading dislocations, twin boundaries, screw dislocations, and misfit dislocations. These linear structures can form continuous pinning pathways when aligned parallel to flux lines, significantly enhancing critical current performance under high magnetic fields with consistent orientation. Particularly, through doping (such as introducing BaZrO_3_ nanorods), artificial one-dimensional pinning arrays with excellent anisotropic pinning capability can be constructed to meet high-field application requirements. Two-dimensional defects consist of grain boundaries, twin planes, stacking faults, anti-phase boundaries, and in-plane local mismatch surfaces [69,70,71]. Distributed within the material’s plane, these defects significantly influence vortex pinning effects, typically existing as planar features at grain interfaces that can obstruct transverse motion of flux lines over large areas, demonstrating remarkable effects in REBCO coated conductors with strong crystalline orientation. The pinning effectiveness of such defects depends on the relative orientation between the magnetic field and defect planes, making important contributions to *J*_c_ enhancement under intermediate to high magnetic fields [72]. Three-dimensional defects include precipitates, voids, or coarse particles as large-scale defects that can provide strong pinning potential barriers in arbitrary directions, exhibiting excellent isotropic characteristics. By engineering the type, distribution, and size of secondary phases, the performance stability of REBCO films under high magnetic fields can be effectively improved [73,74]. Zero-dimensional defects typically involve missing or substituted single atoms or ions, forming point defects or oxygen vacancies that provide localized pinning potential wells at atomic scales. These small-sized, densely distributed defects are suitable for suppressing vortex motion under low-temperature and low-field conditions while demonstrating isotropic pinning properties [70,73]. Although individual defects have limited pinning strength, synergistic effects can emerge at high densities, contributing to overall *J*_c_ enhancement. The roles of pinning centers with different dimensionalities in enhancing superconducting performance are complementary. Rational design and control of multi-scale, multi-dimensional defect structures represent a critical pathway for achieving high-performance REBCO superconducting tapes.

## 5. Enhancement of *J*_c_ Through the Introduction of Artificial Pinning Centers

As previously discussed, achieving high critical current density (*J*_c_)—particularly under strong magnetic fields—requires the effective suppression of vortex motion. Naturally occurring defects in REBCO films, as described in the preceding section, contribute to a certain level of flux pinning. However, the pinning efficiency of these naturally occurring defects is often insufficient to counteract thermal fluctuations, cannot maintain the required *J*_c_ under high applied magnetic fields [75,76]. Consequently, considerable research interest has been directed toward enhancing the *J*_c_ of REBCO thin films by introducing additional defects into the superconductor. Various approaches have been developed for introducing artificial pinning centers (APCs) into REBCO superconductors, which can generally be categorized into three main strategies.

### 5.1. Doping of Rare-Earth Elements (Addition and/or Substitution)

The properties of REBCO high-temperature superconducting thin films are significantly influenced by the type of RE elements (Table 2 [77]) used. Although the rare earth elements primarily occupy the RE sites in the crystal structure and do not directly participate in the formation of superconducting electron pairs, their ionic radii affect the superconducting transition temperature. Early studies demonstrated that the superconducting transition temperature (*T*_c_) varies linearly with the ionic radius of rare earth ions [78], and this phenomenon has been attributed to strain-induced charge redistribution between the charge reservoir (CuO chains) and the CuO_2_ planes [10].

The simplest substitution approach involves replacing Y with other rare earth elements while maintaining the 123 stoichiometric ratios. As mentioned earlier, GdBCO demonstrates significantly better performance than YBCO in magnetic fields, which may be attributed to increased stacking fault density [69]. Studies [79,80] have also reported substantial performance improvements in EuBCO and SmBCO compared to YBCO, though these materials require higher growth temperatures for deposition. Another method for doping rare-earth elements involves mixtures of one or more alternative rare-earth elements while retaining the 123 composition (RE_1_,RE_2_,RE_3_)Ba_2_Cu_3_O_7−δ_. MacManus-Driscoll et al. used PLD to deposit Y_2/3_Sm_1/3_Ba_2_Cu_3_O_7−δ_ films on single-crystal substrates, achieving up to a 3-fold enhancement in *J*_c_ under magnetic fields compared to YBCO [81]. Current research includes (YGdBCO) [82], (EuErBCO) [15], and various Y-Sm mixtures, all of which exhibit performance improvements. It is believed that rare-earth doping enhances flux pinning effects by inducing lattice distortions, thereby improving critical current density. It is worth noting that the creation of mixed rare-earth compounds is not boundless, systematic evaluation is typically conducted using two readily quantifiable parameters: the variance (degree of ion-size difference) and the average ionic radius. Li et al. employed PLD to fabricate a series of (Nd_x_Sm_x_Gd_1−2x_)Ba_2_Cu_3_O_7−δ_ (x = 0, 0.1, 0.25, 0.33) thin films on SrTiO_3_ (STO) single-crystal substrates. The Nd/Sm co-doping introduced point defects, and when x = 0.1, it significantly enhanced the pinning density and *J*_c_ at low temperatures (<20 K). This study confirmed that excessive doping levels degrade the superconducting properties of REBCO films, while moderate doping can effectively improve the in-field *J*_c_ values [83]. Feighan et al. employed liquid-assisted PLD (LAP-PLD) to rapidly grow ~350 nm thick Y_0.8_Yb_0.1_Sm_0.1_BCO + Ba_2_YNbO_6_ (BYNO) films on STO single-crystal substrates using off-stoichiometric targets (RE:Ba:Cu = 1:1.7:2.7). At 10 K and 10 T, *J*_c_ rises from 4.4 to 7.7 MA cm^−2^ compared to YBCO + BYNO, demonstrating that rare-earth co-doping and secondary-phase incorporation act synergistically to dramatically enhance low-temperature, high-field performance [84]. In addition to conventional rare-earth substitution and mixing strategies at the RE sites, a recent study proposed an unconventional approach: partial substitution of Ba sites with rare-earth element Nd. Fujiwara et al. successfully synthesized polycrystalline Nd_1+x_Ba_2−x_Cu_3_O_7−y_ (NdBCO) samples with controlled Nd doping levels (x ≤ 0.05) using a low-oxygen atmosphere sintering method, aiming to investigate the effects of minor Nd substitution at Ba sites on superconducting properties. Through precise control of the Nd/Ba substitution ratio while maintaining single-phase stability, the study observed that the superconducting transition temperature (*T*_c_) increased from 95.0 K to 96.6 K as x varied from 0 to 0.05. The enhanced oxygen content stabilized the overall carrier concentration, indicating that Nd → Ba substitution does not significantly disturb the hole concentration in CuO_2_ planes. This work experimentally demonstrated that appropriate Nd substitution at Ba sites can actually enhance *T*_c_ in NdBCO. The study provides new evidence for understanding the synergistic effects of structure, charge and superconducting properties induced by rare-earth doping at different lattice sites in REBCO crystals, suggesting that material design should more carefully consider “site-specific” effects beyond just elemental species [85].

Rare-earth substitution in REBCO thin films significantly influences their superconducting properties, particularly the critical temperature and current-carrying capability. To provide a clearer comparison, Table 3 summarizes representative REBCO thin films fabricated by PLD in recent years under different substrate conditions and thicknesses, highlighting their *T*_c_ and self-field *J*_c_ at 77 K.

### 5.2. Substrate Surface Decoration

**Figure 6:**
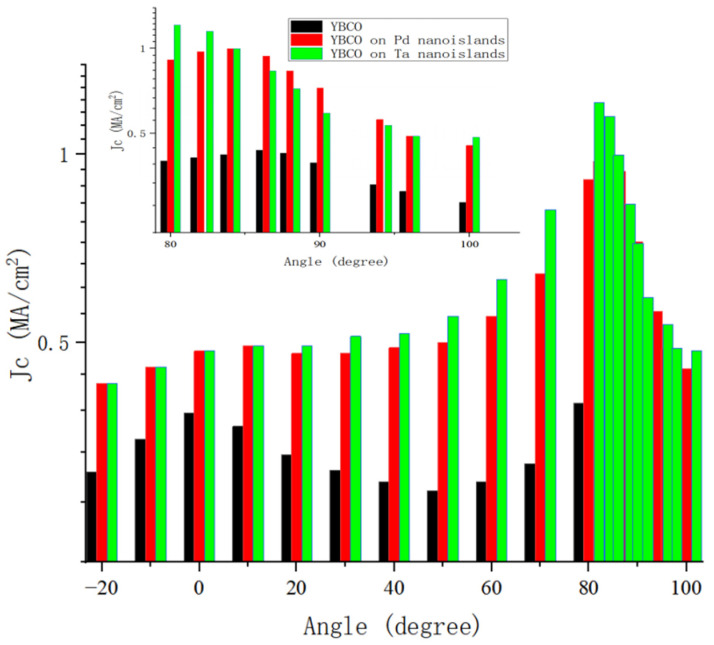
Transport critical current density of 0.8 µm thick YBCO films on Ta- or Pd-decorated LMO cap layers. Dependence of critical current density at 1 T and 77 K on orientation of the applied magnetic field, relative to the film’s normal direction. The data are compared with a similarly prepared YBCO film on an LMO cap layer without any surface decoration.

Substrate surface modification, as a pinning optimization method that does not require altering the superconducting layer process, is one of the earliest approaches used to improve the flux pinning characteristics of REBCO (e.g., YBCO) thin films [69]. Its mechanism involves introducing various metal [101,102] or oxide [103,104,105] nanoparticles on the substrate surface, which generate interfacial defects between the substrate and film, creating grain boundaries and dislocations on the lattice surface of the superconducting phase, thereby inducing correlated or uncorrelated pinning centers during the growth of the superconducting layer to enhance the critical current density under high fields. The B. Maiorov team investigated naturally grown STO particles on the buffer layer surface, demonstrating that the presence of STO particles promotes additional correlated pinning along the c-axis direction in YBCO films, effectively slowing the decay rate of *J*_c_ with magnetic field without requiring additional processes [106]. The research team employed off-axis PLD to grow YBCO films on STO substrates modified with vapor-phase Y_2_O_3_. Experimental results showed that Y_2_O_3_ nanoparticle decoration on single-crystal substrates effectively promotes the formation of APCs and significantly increases *J*_c_ [107]. The Aytug team deposited YBCO on metal substrates decorated with BaTiO_3_ and BaZrO_3_ nanoparticles via PLD, observing a significant improvement in *J*_c_, confirming the universal effectiveness of the artificial nanoparticle surface decoration technique in coated conductors [108]. Jha et al. first deposited ferromagnetic La_0.67_Sr_0.33_MnO_3_ (LSMO) nanodots on STO single crystals using PLD, followed by 400 nm YBCO growth; results showed approximately a 4-fold increase in critical current density at 77 K and 4 T, demonstrating that introducing ferromagnetic nanoparticles on the substrate can effectively enhance the flux pinning effect in YBCO films [109]. Polat et al. employed an industrially compatible DC magnetron sputtering method to pre-deposit Pd or Ta nanoislands on the LaMnO_3_ buffer layer of IBAD metal tapes [110], followed by PLD growth of 0.8 µm YBCO films; compared to unmodified controls, the *J*_c_ decay exponent under c-axis magnetic fields at 77 K decreased from 0.49 to 0.45, with significantly improved high-field *J*_c_, as shown in Figure 6.

**Figure 7:**
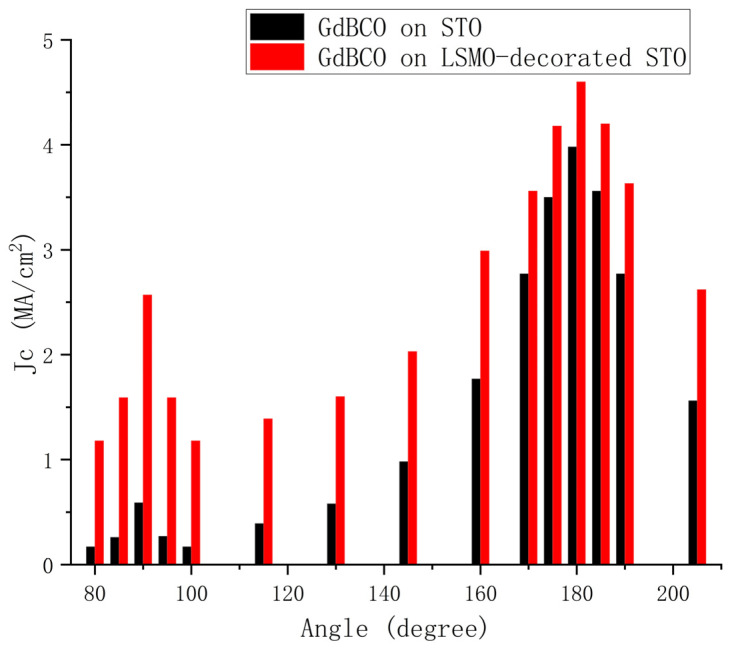
Atomic force microscopic image of LSMO nanoparticle decorated STO substrates: 3D image and Dependence of *J*_c_ at 0.3 T and 77 K on orientation of the applied magnetic field.

In recent years, there have been new developments regarding the mechanism of ferromagnetic LSMO nanoparticle-modified substrates. Wang et al. [111] investigated the influence of LSMO nanoparticles on the pinning characteristics of GdBCO thin films, using RF magnetron sputtering technology to treat the STO substrate surface before growing GdBCO films [109]. Figure 7 shows the image of the STO substrate modified with LSMO nanoparticles. The critical current density *J*_c_ of the modified samples significantly improved compared to unmodified samples, with the pinning force also showing substantial enhancement. Moreover, the magnetic field corresponding to the maximum pinning force (*F*_pmax_) shifted toward higher field values, and the *J*_c_ exhibited notable improvement under different applied magnetic field orientations (Figure 7). This study confirmed that LSMO nanoparticles not only act as pinning centers by inducing threading dislocations, leading to significant *J*_c_ enhancement in the H//c direction, but also establish a magnetic pinning mechanism through their ferromagnetic properties, achieving stronger pinning effects under high-temperature and high-field conditions [111]. This research further validates the potential of substrate surface modification in enhancing high-field pinning characteristics.

Substrate surface modification can also synergize with secondary phases introduced in the superconducting layer to enhance the pinning effect in REBCO films. Rizzo et al. proposed and validated a “synergistic pinning” strategy, which first constructs ZrO_2_ nano-islands on STO single-crystal surfaces via polymer-assisted deposition (PAD), followed by PLD growth of 250 nm YBCO-BYNTO films using composite targets containing 2.5 mol% Ba_2_YNbO_6_ + Ba_2_YTaO_6_ (BYNTO). This approach simultaneously introduces both substrate-modification-induced planar defects and self-assembled BYNTO nanocolumns within the film into a single system [112]. The synergistic pinning mechanism demonstrates more effective conduction channels in the low-temperature-high-field regime, indicating that stacking faults become additional efficient pinning centers in this critical range. Although the nano-islands reduce *T*_c_ by 3.5 K and decrease the self-field *J*_c_, within the crucial operational window of <50 K and >9 T, the synergistic strategy significantly improves pinning efficiency. This provides a novel interface engineering approach for REBCO coated conductors targeting fusion and high-energy physics applications, without requiring modifications to the PLD process.

### 5.3. Introduction of Nanoscale Secondary Phases

**Figure 8:**
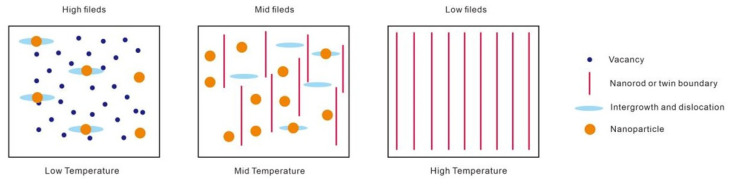
The magnetic field–temperature diagram with three distinguished regions in which vortex physics are governed by defects of different dimensionalities.

Another effective method for introducing APCs is to incorporate nanoscale secondary phases into REBCO superconducting thin films. Studies show these second phases can effectively improve flux pinning while maintaining the film’s structural integrity. The introduction of certain secondary phases contributes to mitigating the intrinsic anisotropy of REBCO superconducting materials—whose superconducting performance (particularly the critical current density, *J*_c_) strongly depends on the orientation of the applied magnetic field relative to the crystal axes—thereby effectively enhancing their isotropic properties. Figure 8 illustrates the vortex pinning phase diagram of REBCO nanocomposites across different temperature and magnetic field regions, revealing how defects of various dimensionalities dominate the vortex pinning mechanism and how their influence evolves with external conditions [113]. In the low-temperature, high-field region, the pinning is mainly governed by a high density of isotropic weak or strong defects, such as atomic and cluster vacancies (0D pinning) and nanoscale strain regions (3D pinning) induced by local tensile strain. These defects suppress the local superconducting order parameter and form stable isotropic pinning potentials, effectively enhancing *J*_c_ under high magnetic fields. As the temperature increases into the intermediate-temperature, intermediate-field region, both strong anisotropic and strong isotropic defects contribute cooperatively. In this regime, long-range coherent nanorods (1D pinning), twin boundaries, and stacking faults (2D pinning) work together with nanoparticles (3D pinning) to form a hybrid pinning landscape, which provides continuous vortex anchoring along the c-axis while simultaneously enhancing lateral vortex stability. This hybrid structure produces balanced pinning strength across different magnetic field orientations and suppresses flux creep. Entering the high-temperature region, near the irreversibility line *B*_irr_(*T*), long anisotropic strong defects—such as extended twin boundaries and vertically aligned nanorods—dominate the pinning landscape and sustain vortex immobilization at elevated temperatures. However, due to significant thermal activation, relying solely on anisotropic defects leads to flux creep and weakened pinning. Thus, incorporating strong isotropic defects such as small nanoparticles and nanostrain regions creates a composite or hybrid pinning structure that improves thermal stability and maintains high *J*_c_. Overall, the cooperation and competition among 0D, 1D, 2D, and 3D defects together define the optimal pinning landscape and performance window of REBCO nanocomposites under various operational environments.

With PLD emerging as one of the most industrially promising strategies for introducing secondary phases. Noble metals like silver, gold, palladium, or platinum [114,115,116] have been widely demonstrated to induce strong pinning in REBCO due to their chemical inertness and high conductivity, though their high costs and resource scarcity have driven researchers toward more engineering-feasible secondary phase systems. Based on crystal structure, chemical compatibility, and functional differences, these secondary phases mainly fall into the following categories: The first category encompasses multifunctional oxides with ferromagnetic/non-magnetic/insulating properties, such as LSMO, STO, or CeO_2_ [117]. Beyond their use as pre-deposited nanoislands on substrates mentioned previously, they can also be introduced through co-sintered targets to form island-column-stacking fault synergistic networks, combining magnetic pinning, strain pinning, and interface energy modulation effects. The second category includes rare-earth oxides RE_2_O_3_ (Y_2_O_3_, Gd_2_O_3_, etc.) [117,118], which tend to form equiaxed precipitates due to their high interfacial energy, inducing high-density stacking faults that provide planar defects. The third category consists of perovskite or double-perovskite families with lattice mismatches below 7%, representing the current research focus for secondary phases. Typical examples include BaZrO_3_ (BZO), BaHfO_3_ (BHO), BaSnO_3_ (BSO), and Ba_2_Y(Nb/Ta)O_6_ [117,119,120], which self-assemble into c-axis-aligned nanocolumns penetrating the film, effectively enhancing *J*_c_ in high-field environments. Another important secondary phase system is RE2BaCuO5 (RE211), such as Y211 and Gd211 [121,122]. As a rare-earth barium oxide, RE211 plays a significant role in pinning due to its excellent crystal structure and chemical compatibility with REBCO. RE211 not only helps improve the material’s flux pinning effect but can also optimize superconducting performance under high fields and temperatures by controlling its morphology and distribution. Through optimization and improvement of secondary phases and PLD processes, high-performance REBCO tapes can now be produced in batches [1,113,117], laying a solid material foundation for REBCO conductors’ high-field applications in fusion magnets, accelerators, and compact NMR systems.

**Figure 9:**
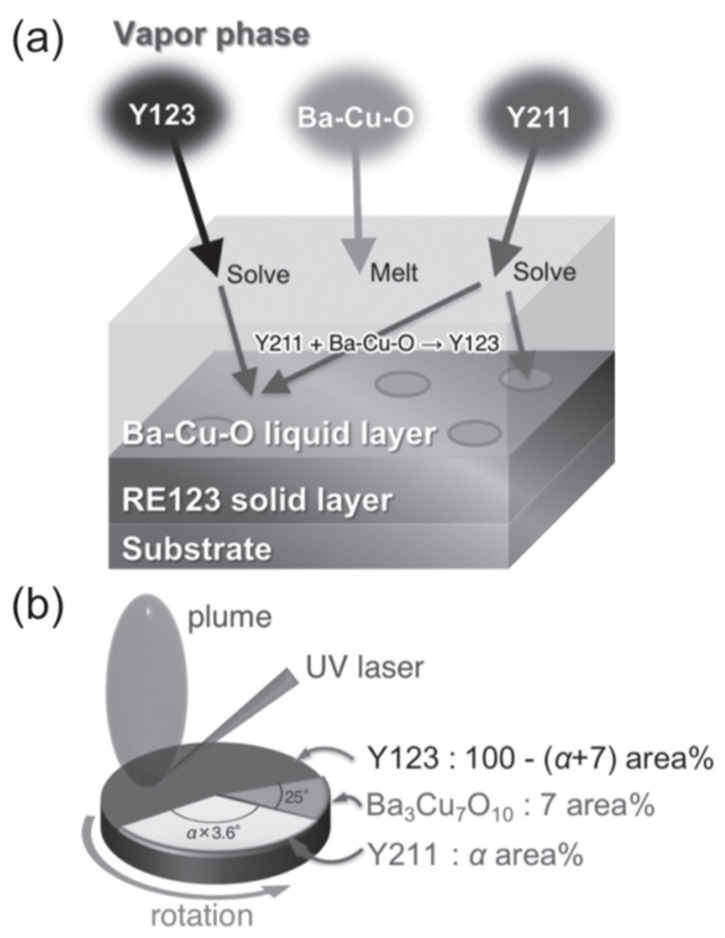
Schematic drawing of (**a**) VLS growth method and (**b**) surface modified target for Y211-doped VLS-Y123 films [125].

**Figure 10:**
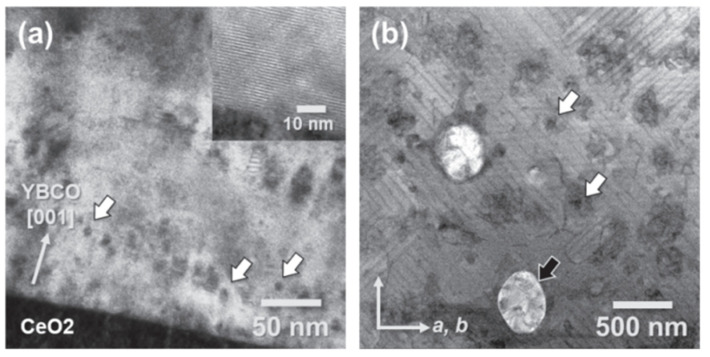
(**a**) Cross-sectional and (**b**) plan-view TEM images of Y211-doped VLS-YBCO film (α = 25 area%) [125].

**Figure 11:**
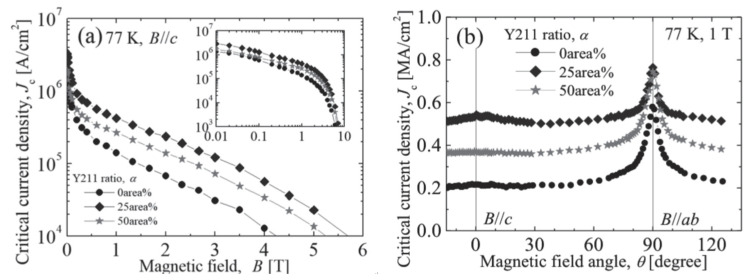
(**a**) Magnetic field dependence and (**b**) angular dependence of *J*_c_ in VLS-YBCO films with various Y211 doping level.

**Figure 12:**
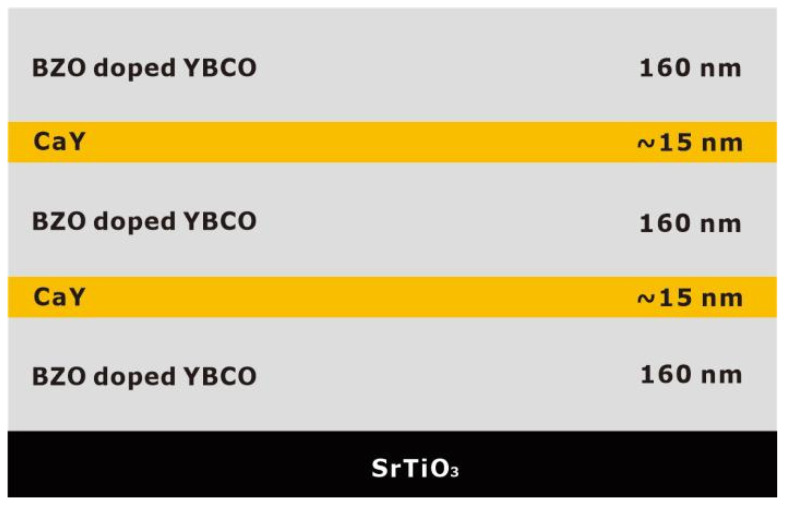
Structural schematic of a 500 nm thick, 6% BZO-doped YBCO multilayer film (containing two CaY-123 layers).

The introduction of nanoparticles represents one of the effective approaches for constructing efficient pinning centers. Wang et al. employed reel-to-reel PLD technology to prepare YBCO films doped with 1–3 mol% CeO_2_ on metal substrates. The 1 mol% doped sample exhibited approximately 33% higher *I*_c_ compared to pure YBCO [24]. The study demonstrated that moderate CeO_2_ doping refined grain size and reduced high-angle grain boundaries, while excessive doping decreased *I*_c_ due to lattice distortion, with the first observation of in-plane grain rotation induced by low-temperature deposition. In earlier studies, the research teams of Haugan [123] and Barnes [124] successively used pulsed laser deposition technology with alternating deposition methods to prepare YBa_2_Cu_3_O_7−δ_/Y_2_BaCuO_5_ (Y123/Y211) multilayer films on single crystals, introducing Y_2_BaCuO_5_ (Y211) nanoparticles into the multilayers. This approach enhanced the critical current density of YBa_2_Cu_3_O_7−δ_ films by 2–3 times at 77 K under 1 T magnetic field. Jha et al. utilized PLD technology with composite targets to prepare YBCO + Y211 nanocomposite films on STO substrates, achieving uniform dispersion of 4–10 nm Y211 nanoparticles within the Y123 matrix. This resulted in isotropic enhancement of *J*_c_ across all angles at 77 K under 1 T magnetic field, with the irreversible field increasing from 7.8 T to 9.7 T [121]. Tsuchiya et al. developed a vapor–liquid–solid (VLS) hybrid growth method (Figure 9) to prepare YBCO films with different Y211 doping ratios on metal substrates. Using modified target methods (as shown in Figure 10, they introduced 6–9 nm Y211 nanoparticles into the films (Figure 11). At a doping ratio of 25%, the self-field *J*_c_ reached 2.9 MA cm^−2^ at 77 K, three times that of undoped films, with isotropic performance improving by 2.6 times at 77 K under 1 T magnetic field (Figure 11) [125]. From multilayer alternating deposition to direct preparation of composite films, the continuous refinement in controlling the size and distribution of Y211 nanoparticles has significantly improved the flux pinning and high-field current-carrying performance of YBCO films. The morphological characteristics are pivotal because they directly influence the efficacy of flux pinning and the behavior of vortex dynamics [32,126,127]. The incorporation of BZO nanoscale inclusions into YBCO films has been implemented in multiple studies, including investigations on further enhancing the pinning performance of YBCO:BZO films [128] and research on the optimal concentration of BZO inclusions for field-dependent pinning effects [129]. The approximately 7.7% lattice mismatch between BZO and YBCO creates interfacial defects that limit pinning enhancement. To overcome this bottleneck, researchers [130,131,132] introduced Ca_0.3_Y_0.7_Ba_2_Cu_3_O_7−x_ (CaY-123) interlayers as Ca sources during PLD deposition. Utilizing a strain-guided Ca diffusion mechanism, this approach induces Ca/Cu substitution at BZO/YBCO interfaces, dynamically expanding YBCO’s c-axis lattice parameter and reducing the lattice mismatch to about 1.4%, thereby significantly minimizing interfacial defects. This multilayer architecture (Figure 12) effectively enhances both pinning force density (*F*_pmax_) and *J*_c_ across the 2–8% BZO doping range while markedly reducing *J*_c_ anisotropy [133]. Aye et al. systematically investigated the influence of BZO nanorod density in YBCO films, demonstrating that at approximately 10 wt% BZO content—where nanorod spacing approaches their diameter—the flux pinning force reaches its maximum, with high-field *J*_c_ substantially outperforming lightly doped samples. However, excessive doping causes nanorod misalignment and crystalline degradation, ultimately degrading performance [134].

Researchers have employed PLD to introduce double-perovskite structured Ba_2_YTaO_6_ (BYTO), Ba_2_YbNbO_6_ (BYNO), and Ba_2_LuNbO_6_ (BLNO) [20,135] into REBCO thin films as pinning centers. These REBCO films exhibit outstanding flux pinning performance at 77 K, with their irreversible fields significantly exceeding 10 T [136]. By controlling the PLD growth rate, Celentano et al. successfully modified the morphology of double-perovskite nanocolumns in YBCO-BYNTO films from straight and dense to curved and short, achieving high pinning forces of 13.5 GN m^−3^ at 77 K and 900 GN m^−3^ at 4.2 K, thereby realizing optimal synergistic flux pinning performance across the entire temperature range [137].

BHO, with its relatively small lattice mismatch (≈6.7%), outperforms BZO as a typical APC. When introduced into REBCO thin films via PLD, it self-assembles into nanoparticles or short nanorods, significantly enhancing the *J*_c_ and pinning force (*F*_p_) under low-temperature and high-field conditions [70,72,138]. Its isotropic pinning characteristics surpass those of traditional nanocolumns, garnering widespread attention from researchers. Ichino et al. achieved precise control over the size and density of BHO nanocolumns by regulating deposition temperature (790–880 °C) through low-temperature growth (LTG) technology [139]. Studies indicate that fine-diameter, high-density BHO nanocolumns are ideal artificial pinning centers for high-field applications [140]. When introducing 5 mol% BHO doping during the preparation of YGBCO superconducting films using seed layer technology, *J*_c_ was further improved without reducing *T*_c_ [50]. Some studies [141,142] have successfully prepared high-performance BHO-doped EuBCO coated conductors by modifying the PLD growth process (VLS-PLD). The team led by Ibi [96] fabricated high-performance REBCO films with high-concentration BHO doping through high-temperature deposition and low-temperature oxygen annealing processes. Using ultra-high-speed dynamic MPMT-PLD, layered-tunable BHO defects were constructed in EuBCO for the first time. The 6% BHO-doped EuBCO film exhibited an extremely high pinning force density (*F*_p_ ≈ 9 × 10^2^ GN m^−3^) at 4.2 K and 14 T with the magnetic field parallel to the c-axis [96]. This study established a quantitative correlation between defect morphology and pinning mechanisms, providing a replicable process paradigm for defect engineering in industrial-grade high-field REBCO coated conductors.

Compared to introducing a single secondary phase, researchers are now shifting towards co-doping multiple secondary phases in REBCO tapes. The mixed pinning strategy employing various types of pinning centers is considered more effective in achieving the dual goals of high *J*_c_ values and reduced *J*_c_ anisotropy [120,143,144]. Through a hybrid pinning approach combining BHO nanocolumns and Y_2_O_3_ nanoparticles [131], the flux pinning force *F*_p_ of YBCO reached 1.57 TN/m^3^ at 4.2 K, as high as the record *F*_p_, max levels reported at that time in the (Gd,Y)BCO + BaZrO_3_ system and SmBCO + BHO (1.6 TN m^−3^) [145]. This BHO + Y_2_O_3_ co-doping strategy also significantly reduced the anisotropy of *J*_c_. In the latest research, a team proposed and implemented a “BaSnO_3_ + BaHfO_3_” dual-phase co-doping strategy based on the MPMT-PLD reel-to-reel system. Under high-speed deposition conditions [146], this approach self-assembled a network of BaSnxHf1−xO_3_ (BSHO) nanocolumns with diameters of about 5 nm and an areal density as high as 2200 μm^−2^. The formation mechanism of these BSHO solid solutions originates from the atomic-scale mutual substitution of Sn^4+^ and Hf^4+^ cations within the perovskite lattice during ultra-high-rate PLD. Because Sn and Hf possess similar ionic radii and identical valence states, they can readily occupy the B-site of the BaMO_3_ perovskite lattice, forming a homogeneous solid solution instead of separate BaSnO_3_ or BaHfO_3_ phases. Under the limited surface diffusion time associated with deposition rates exceeding 100 nm/s, Sn and Hf atoms nucleate simultaneously and coalesce into mixed BSHO nanocolumns. The higher diffusivity of Sn promotes the formation of numerous nuclei, increasing the nanocolumn density, while the lower diffusivity of Hf restricts excessive columnar growth, resulting in uniform, fine, and highly aligned nanocolumns. The BSHO nanocolumns establish semi-coherent interfaces with the EuBCO matrix, where alternating Ba–O: Hf/Sn–O bonding and edge dislocations accommodate lattice mismatch. This strain-driven interface formation stabilizes the BSHO phase and generates numerous nanoscale defects that act as additional random pinning centers under high magnetic fields. This unique nanostructure significantly enhanced the *J*_c_ of co-doped EuBCO films under applied magnetic fields. At 50 K and 3 T with the magnetic field parallel to the c-axis, *J*_c_ reached approximately 4.0 MA/cm^2^, exceeding the parallel field value. Even under an 8T perpendicular field, the *J*_c_(θ) curve still exhibited significant pinning peaks, outperforming mainstream commercial tapes from Fujikura, SuperPower, and Shanghai Superconductor. At 4.2 K in high fields, the pinning force showed non-saturation behavior, reaching a high value of about 990 GN/m^3^ up to 24 T, significantly surpassing similar materials prepared by MOCVD (α index 0.77 compared to >0.9). This co-doping strategy of BSO and BHO effectively improved the in-field performance of EuBCO films, particularly their pinning capability under high magnetic fields and low temperatures, demonstrating the tremendous potential of this method for practical superconducting applications.

The incorporation of nanoscale secondary phases into REBCO thin films has been demonstrated as an effective strategy to enhance flux pinning and improve high-field performance, and it currently represents a key research focus in coated conductor development. Table 4 summarizes recent advances on REBCO thin films modified with different types of secondary phases, presenting their critical temperatures and critical current density performance under various temperature and magnetic field conditions.

## 6. Conclusions

In summary, REBCO coated conductors have become the cornerstone material for high-field applications, where performance optimization relies on the synergy between fabrication techniques and microstructural engineering. PLD with its accurate stoichiometric transfer, excellent epitaxial growth capability, and versatile process control, has emerged as a leading method for producing high-quality REBCO films. By optimizing parameters such as laser fluence, substrate temperature, oxygen partial pressure, and annealing conditions, films with excellent crystallinity and high *J*_c_ can be obtained. At the same time, the incorporation of APCs is critical for enhancing *J*_c_. These include lattice distortions introduced by rare-earth doping, interfacial defects induced by substrate surface modification, and nanoscale secondary phases (e.g., BZO, BHO, RE_2_O_3_) that construct multi-scale defect networks. The combined application of these approaches provides a viable pathway to achieving high-performance REBCO coated conductors.

Current research has moved beyond single pinning strategies toward synergistic pinning mechanisms, as well as from laboratory-scale samples to long-length industrial fabrication. Balancing high *J*_c_ with uniformity, anisotropy control, and cost-effectiveness remains a central challenge. Looking forward, several directions are particularly important:

1. Compatibility of high-throughput PLD with complex pinning architectures—enabling precise control of nanostructures while maintaining high deposition rates.
2. Integration of multiple fabrication routes—combining PLD with techniques such as MOCVD and MOD to optimize both performance and cost.
3. Exploration of novel secondary phases—future studies should focus on the discovery and controlled incorporation of unconventional secondary phases beyond conventional perovskite oxides and rare-earth oxides. The development of novel nanostructured compounds or heterostructures with enhanced chemical compatibility and tunable functionalities may enable new flux pinning mechanisms, thereby offering additional opportunities to further improve the in-field performance of REBCO coated conductors.
4. Further optimization of reel-to-reel technologies—validating process stability and uniformity in kilometer-scale conductors.

Overall, REBCO coated conductor research is increasingly application-driven, and future breakthroughs will likely arise from interdisciplinary collaboration and innovations in process engineering.

## Figures and Tables

**Table 1:** Technology routes, composition, and performance for international industrial partners, as well as electrical measurement methods [19,40,41,42,43,44,45].

Company	Technical Route	Substrate	Buffer Layer	*I*_c_@77 K, Self-Field (A/cm-w)
SuperPower (Schenectady, NY, USA)	IBAD/MOCVD-REBCO	Hastelloy	Al_2_O_3_/Y_2_O_3_/MgO/LaMnO_3_	400
Fujikura (Tokyo, Japan)	IBAD/PLD-GdBCO	Hastelloy	Al_2_O_3_/Y_2_O_3_/MgO/CeO_2_	725
Shanghai Creative Superconductor Technology Corporation (Shanghai, China)	IBAD/MOD-REBCO	Hastelloy	Al_2_O_3_/Y_2_O_3_/MgO/LaMnO_3_	900
Shanghai Superconductor Technology Corporation (Shanghai, China)	IBAD/PLD-REBCO	Hastelloy	Al_2_O_3_/Y_2_O_3_/MgO/CeO_2_	600
SuNAM (Daejeon, Republic of Korea)	IBAD/RCE-GdBCO	Hastelloy	Al_2_O_3_/Y_2_O_3_/MgO/LaMnO_3_	700
Eastern Superconductor Technology Corporation (Suzhou, China)	IBAD/MOCVD-REBCO	Hastelloy	Al_2_O_3_/Y_2_O_3_/MgO/LaMnO_3_	>300
Faraday Factory Japan (Sagamihara, Japan)	IBAD/PLD-REBCO	Hastelloy	Al_2_O_3_/Y_2_O_3_/MgO/LaMnO_3_	585
Supermag Technology (Shanghai, China)	IBAD/PLD-REBCO	Hastelloy	Al_2_O_3_/Y_2_O_3_/MgO/CeO_2_	>500

**Table 2:** Ionic radii (trivalent, 8 coordination) of rare earth (RE) elements of REBCO superconductor [77].

RE Element	Tm^3+^	Yb^3+^	Er^3+^	Dy^3+^	Ho^3+^	Y^3+^	Gd^3+^	Eu^3+^	Sm^3+^	Nd^3+^
Ionic radius (Å)	0.99	0.99	1.00	1.00	1.02	1.04	1.05	1.07	1.08	1.12

**Table 3:** Critical temperature and self-field critical current density at 77 K of REBCO thin films with different rare-earth substitutions.

Film	Film Deposition Process and Substrate	Thickness (µm)	*T*_c_ (K)	*J*_c_ @ Self-Field, 77 K (MA cm^−2^)	Reference (Ref.)
YGdBCO	multi-channel and multi beam PLD, C276/Al_2_O_3_/Y_2_O_3_/IBAD-MgO/CeO_2_	2.9		1.44	[86]
HoGdBCO	multi-channel and multi beam PLD, C276/Al_2_O_3_/Y_2_O_3_/IBAD-MgO/CeO_2_	0.9		3.9	[86]
GdBCO	PLD, MgO single crystals	0.22–0.25	92.7	3.2	[87]
YBCO	PLD, SrTiO_3_/LaMnO_3_	0.45	90.0	1.45	[88]
YBCO	reel-to-reel PLD, stainless steel/YSZ/CeO_2_	0.7	90.5	2.7	[89]
GdBCO	reel-to-reel PLD, Hastelloy/Al_2_O_3_/Y_2_O_3_/IBAD-MgO/ LaMnO_3_/CeO_2_		93.3	3.18	[90]
GdBCO	PLD, Hastelloy/Al_2_O_3_/Y_2_O_3_/IBAD-MgO/ LaMnO_3_/CeO_2_	2		3	[91]
Nd_0.1_Sm_0.1_Gd_0.8_BCO	PLD, STO single crystals		92.0	2.5	[92]
NdErGdBCO	PLD, MgO single crystals	0.5	92.5	3.18	[93]
DyHoBCO	PLD, LAO single crystals	0.5–0.6	90.3	1.25	[94]
SmBCO	PLD, Hastelloy/Gd_2_Zr_2_O_7_/Y_2_O_3_/IBAD-MgO/LaMnO_3_/CeO_2_	0.5	93.1	3.0	[95]
EuBCO	reel-to-reel PLD Hastelloy/Gd_2_Zr_2_O_7_/Y_2_O_3_/IBAD-MgO/LaMnO_3_/CeO_2_	0.5–0.55	93.4	5.7	[96]
NdBCO	PLD, STO single crystals		91.2	3.5	[97]
TmBCO	PLD, STO single crystals	0.062	86	4.5	[98]
ErBCO	PLD, STO single crystals		89.7	1.0	[99]
HoBCO	PLD, Ni-alloy/CeO_2_/YSZ/CeO_2_	0.2	~90	5.6	[100]

**Table 4:** Critical temperature and critical current density of REBCO thin films modified with different secondary phases under various conditions.

Film	Introduction of Nanoscale Secondary Phases and Dimension	Film Deposition Process and Substrate	Thickness (µm)	Tc (K)	*J*_c_ (MA cm^−2^)			Ref
					High-Field, Low Temperature	Mid-Field, Mid Temperature	Self-Field, 77 K	
YBCO	CeO_2_ nanoparticles (3D)	reel-to-reel PLD, C276/Al_2_O_3_/Y_2_O_3_/IBAD-MgO/CeO_2_	0.2				5.0	[24]
YBCO	Y_2_O_3_ nanoparticles (3D)	PLD, STO single crystals	0.28	90.0			3.6	[147]
GdBCO	Gd_2_O_3_ nanoparticles (3D)	PLD, MgO single crystals	0.23	93.3	3.66 (10 K, 8 T) 2.61 (10 K, 14 T)		3.7	[87]
YBCO	Y211 nanoparticles (3D)	PLD, STO single crystals		89.6		0.93 (65 K, 3 T) 0.49 (65 K, 7 T)	2.3	[121]
YBCO	Y211 nanoparticles (3D)	VLS, Hastelloy/Gd_2_Zr_2_O_7_/Y_2_O_3_/IBAD-MgO/LaMnO_3_/CeO_2_	0.4	91.3			2.9	[125]
YBCO	BLNO nanocolumns (1D)	PLD, STO single crystals	0.16	90.0			0.7	[135]
SmBCO	BSNO nanocolumns (1D)	PLD, LAO single crystals	0.5	90.4		~1.3 (65 K, 0.8 T)	1.1	[148]
SmBCO	BHO nanocolumns (1D)	PLD, LAO single crystals	0.26	91.5	18.06 (4.2 K, 8 T) 11.29 (4.2 K, 14 T)		2.1	[149]
(Eu,Er)BCO	BHO nanocolumns (1D) and irradiation-induced nanoparticles (3D)	reel-to-reel PLD Hastelloy/Gd_2_Zr_2_O_7_/Y_2_O_3_/IBAD-MgO/LaMnO_3_/CeO_2_	0.5	91.7		2.20 (65 K, 3 T) 1.32 (65 K, 6 T)	4.89	[15]
EuBCO	BHO nanocolumns (1D) and nanoparticles (3D)	reel-to-reel MPMT-PLD (facility), C276/Al_2_O_3_/Y_2_O_3_/IBAD-MgO/LaMnO_3_/CeO_2_	1.1	96.9	9.1 (4.2 K, 8 T) 5 (4.2 K, 14 T)	2.8 (50 K, 3 T)	2.59	[150]
EuBCO	BHO nanocolumns (1D) and nanoparticles (3D)	reel-to-reel MPMT-PLD (facility), C276/Al_2_O_3_/Y_2_O_3_/IBAD-MgO/LaMnO_3_/CeO_2_	3.3	97.2	8.6 (4.2 K, 8 T) 5 (4.2 K, 14 T)	2.6 (50 K, 3 T)	1.39	[150]
YBCO	BHO nanocolumns (1D)	VLS, Hastelloy/Al_2_O_3_/Y_2_O_3_/IBAD-MgO/LaMnO_3_/CeO_2_		90.0		0.37 (65 K, 3 T) 0.13 (65 K, 7 T)	0.9	[142]
Sm_1.075_Ba_1.925_Cu_3_O_y_	BHO nanocolumns (1D)	PLD, Hastelloy/Al_2_O_3_/Y_2_O_3_/IBAD-MgO/CeO_2_	0.2	90.4			5.1	[151]
YBCO	BCO nanoparticles (3D)	PLD, STO single crystals	0.16	86.0	4.66 (10 K, 6 T)	1.88 (40 K, 4 T) 1.74 (40 K, 6 T)		[152]
GdBCO	BSO nanocolumns (1D)	reel-to-reel PLD, Hastelloy/Al_2_O_3_/Y_2_O_3_/IBAD-MgO/LaMnO_3_/CeO_2_		92.0	6.94 (4.2 K, 8 T)		2.05	[90]
YBCO	BZO nanocolumns (1D)	PLD, STO single crystals	0.2	89.0	3.65 (10 K, 8 T)	3.25 (40 K, 4 T) 2.31 (50 K, 8 T) 1.16 (65 K, 8 T)		[153]
YBCO	BYNTO nanocolumns (1D) and Y_2_O_3_ nanoparticles (3D)	PLD, STO single crystals	0.15–0.23	88.0		1.8 (65 K, 3 T) 0.88 (65 K, 5 T)	3	[136]
YBCO	BHO nanocolumns (1D) and Y_2_O_3_ nanoparticles (3D)	PLD, STO single crystals	0.16–0.20	88.5	5.32 (5 K, 8 T)	2.57 (50 K, 3 T) 1.07 (50 K, 6 T) 0.9 (65 K, 3 T)	3.7	[154]
EuBCO	BSHO nanocolumns (1D)	reel-to-reel Ultra-high Rate PLD (facility), Hastelloy/Al_2_O_3_/Y_2_O_3_/IBAD-MgO/LaMnO_3_/CeO_2_	1	95.81	9.62 (4.2 K, 8 T) 6.32 (4.2 K, 14 T)	4 (50 K, 3 T)	2.6	[146]

## Data Availability

Data sharing is not applicable.
